# Darwin’s sexual selection hypothesis revisited: Musicality increases sexual attraction in both sexes

**DOI:** 10.3389/fpsyg.2022.971988

**Published:** 2022-08-25

**Authors:** Manuela M. Marin, Ines Rathgeber

**Affiliations:** ^1^Department of Cognition, Emotion and Methods in Psychology, University of Vienna, Vienna, Austria; ^2^Department of Psychology, University of Innsbruck, Innsbruck, Austria

**Keywords:** origins of music, cross-modal priming, sexual selection, face perception, romantic attraction, mate choice, evolutionary musicology

## Abstract

A number of theories about the origins of musicality have incorporated biological and social perspectives. Darwin argued that musicality evolved by sexual selection, functioning as a courtship display in reproductive partner choice. Darwin did not regard musicality as a sexually dimorphic trait, paralleling evidence that both sexes produce and enjoy music. A novel research strand examines the effect of musicality on sexual attraction by acknowledging the importance of facial attractiveness. We previously demonstrated that music varying in emotional content increases the perceived attractiveness and dating desirability of opposite-sex faces only in females, compared to a silent control condition. Here, we built upon this approach by presenting the person depicted (target) as the performer of the music (prime), thus establishing a direct link. We hypothesized that musical priming would increase sexual attraction, with high-arousing music inducing the largest effect. Musical primes (25 s, piano solo music) varied in arousal and pleasantness, and targets were photos of other-sex faces of average attractiveness and with neutral expressions (2 s). Participants were 35 females and 23 males (heterosexual psychology students, single, and no hormonal contraception use) matched for musical background, mood, and liking for the music used in the experiment. After musical priming, females’ ratings of attractiveness and dating desirability increased significantly. In males, only dating desirability was significantly increased by musical priming. No specific effects of music-induced pleasantness and arousal were observed. Our results, together with other recent empirical evidence, corroborate the sexual selection hypothesis for the evolution of human musicality.

## Introduction

Although music, like language, is a universal phenomenon (Blacking, 1973; Brown, 1991), its origins remain unclear, especially because musical behavior has no apparent immediate survival value (Darwin, 1871). Fitch (2015, 1) has argued that the debate about the origins of music can only move forward fruitfully if the object of study is clearly defined. He suggests differentiating between *musicality* (i.e., “the set of capacities and proclivities that allows our species to generate and enjoy music in all of its diverse forms”) and *music* (i.e., “the product of human musicality”). For example, studying humans’ ability to sing may offer insights into the basis of human musicality, whereas studying the diverse forms and social functions of songs across cultures may be an endeavor followed by ethnomusicologists. Both approaches can mutually support each other. Musicality as a stable human trait can be discussed within a bio-musicological framework (Wallin, 1991) and studied from cognitive, developmental, neural, comparative (Fitch, 2015) as well as evolutionary (Wallin, 1991) perspectives. Varella et al. (2017, 758) define the broader term of *artisticality* as “the instinctive propensities to develop psychological faculties that underlie a whole array of multimodal and extraordinary aesthetically enhancing activities,” which encompasses musicality among other things.

### Sexual selection and the evolution of musicality

The current debate about the origins of music involves a wide range of theories, which can be broadly categorized into adaptationist (Huron, 2001; Honing and Ploeger, 2012) and non-adaptationist (James, 1890; Sperber, 1996; Pinker, 1997). Darwin’s sexual selection hypothesis of the evolution of musicality (Darwin, 1871) is one of the three frequently discussed (e.g., Zahavi and Zahavi, 1999; Miller, 2000; Varella et al., 2017; Verpooten, 2021) – and not mutually exclusive – adaptationist theories of the origins of musicality, alongside proposals for a prominent role of music in social cohesion (e.g., Roederer, 1984; Dunbar, 2004; Brown, 2007; Savage et al., 2020), parental care and infant communication (e.g., Dissanayake, 2000, 2008; Falk, 2004; Mehr et al., 2020; Leongómez et al., 2021) and territorial antipredatory and territorial defense (e.g., Hagen and Hammerstein, 2009) through natural selection. The present study aims to further elucidate the role of sexual selection in the evolution of musicality from a psychological perspective.

Falsification, and not speculation, is at the core of any theory building (Popper, 1935), which will ultimately be the only way to shed light on the complexity of human musicality and its origins. Here, we thus aim to empirically test Darwin’s sexual selection hypothesis of music (Darwin, 1871; Miller, 2000, see also Bannan, 2017). Darwin, comparing music to reproductive behavior in animals (such as in birds, insects, amphibians, fish, reptiles and gibbons), argued that music acts as a courtship display in reproductive partner choice. As a costly and “honest” signal of fine motor skills (Miller, 2000) and advanced cognitive abilities (Charlton, 2014) musicality may be indicative of biological fitness and good genes and thus the result of selection pressure in the social environment (for a review see Karamihalev, 2013; Ravignani, 2018).

Darwin did not regard musicality as a sexually dimorphic trait, which is in consonance with both males and females producing and enjoying music. However, sex differences regarding musicality in adulthood have been documented (e.g., Sluming and Manning, 2000; Varella et al., 2010; Miles et al., 2016). Darwin wrote (1871, 572), “I conclude that musical notes and rhythm were first acquired by the male or female progenitors of mankind for the sake of charming the opposite sex.” This does not preclude the possibility of sex differences in musicality and mating behavior, such as those observed related to the human voice (e.g., Evans et al., 2008; Valentova et al., 2017, 2019; Pisanski et al., 2018), which Darwin (1871, 573) already noted: “Women are generally thought to possess sweeter voices than men, and as far as this serves as any guide, we may infer that they first acquired musical powers in order to attract the other sex.” Darwin also proposed a common origin of music and language, known as the musical protolanguage hypothesis, for which some empirical evidence has emerged (Thompson et al., 2012). This theory argues for a common ancestor of music and speech.

Some evidence for a genetic basis for musicality, a necessary prerequisite for adaptation (Croston et al., 2015), has accumulated (Gingras et al., 2015; Mosing et al., 2015; Järvelä, 2018; Beccacece et al., 2021). In general, heritability depends on the specific aspect of musicality under consideration. For example, heritability estimates seem to be larger for pitch perception than for rhythm perception abilities (Pulli et al., 2008; Seesjärvi et al., 2015). Musical development (i.e., the enculturation into a musical system) appears to follow a standard developmental schedule as well (Hannon and Trainor, 2007). However, although musicality has a moderate genetic basis, musical ability and mating success were negatively associated in a twin study (Mosing et al., 2015). Further evidence against the sexual selection hypothesis of music includes the finding that musicians and non-musicians reported similar sexual activity (Harrison and Hughes, 2017).

From a psychological perspective, it is a challenging task to develop experimental paradigms that can offer insights into musicality’s evolutionary roots. Several experiments have provided mixed empirical support for Darwin’s assertion so far. For example, visually displaying a musical instrument may increase male attractiveness in social media (Tifferet et al., 2012), whereas attractiveness ratings do not differ for fictitious verbal profiles of musicians and non-musicians, neither in males nor females (Bongard et al., 2019). These studies did not directly examine the effect of music experience (i.e., musical sounds) on sexual attraction and courtship (Darwin, 1871), which seems to be more relevant in the context of Darwin’s theory. Two studies by Charlton et al. (2012) and Charlton (2014) involving actual music examined the effect of fertility cycle phase on mate choice in groups of females. First, it was shown that complex music was preferred to simple music, but there was no effect of cycle phase (Charlton et al., 2012). In a follow-up two-alternative forced-choice task (Charlton, 2014), females were told that the two musical excerpts were composed by male composers. Females in the fertile phase of their cycle preferred complex excerpts to simple ones, but only when the composers were potential short-term – and not long-term – sexual partners. This is in line with Darwin’s theory that females may gain genetic benefits for offspring by selecting partners that show musical proficiency.

### Multiple cues in mate choice

Another line of research has started to investigate the effect of music on sexual attraction by considering other relevant biological cues in mate choice, such as the human face (May and Hamilton, 1980; Marin et al., 2017; Madison et al., 2018). It is very likely that in evolutionary history multiple cues of various modalities have played a role in romantic attraction (Valentova et al., 2017, 2019), especially in a small-group social setting, in which music and dance were probably performed and enjoyed (Morley, 2013; Killin, 2018; for a review of human dance and evolution see Fink et al., 2021). The human face is the main factor determining physical attractiveness regarding short- and long-term relationships because it indicates genetic fitness (Currie and Little, 2009), but cognitive factors such as creativity and intelligence may also play a significant role (Boogert et al., 2011). This research approach is backed up by previous findings from the crossmodal priming and multimodal interaction literature, suggesting that sound and music can alter the perception of various types of visual stimuli, including facial expressions (Logeswaran and Bhattacharya, 2009, for a review see Gerdes et al., 2014). Likewise, music performance research has shown that visual information related to the performer can also alter the appreciation of music (Platz and Kopiez, 2012). In short, combining multiple cues in an experimental design enhances ecological validity because music was, until the last century, always perceived live in a multimodal setting, with the performer being present.

Watkins (2017) demonstrated that overall ratings of males, and females’ attractiveness depend on facial attractiveness and creative story-telling ability. A follow-up study involving a divergent thinking measure showed that males’ overall attractiveness was similarly rated when less attractive faces were paired with creative texts and attractive faces with less creative texts, suggesting a compensating interplay between physical and cognitive factors. This effect was not observed for ratings of female attractiveness, for which facial attractiveness was the main determinant of their overall perceived attractiveness. Madison et al. (2018) conducted a similar study by combining displays of three levels of musical creativity (i.e., improvisations of different quality) with three levels of facial attractiveness. Male and female participants, while listening to the music, rated four mate value scales (intelligence, health status and parenting skill) and four mate preference scales (date, intercourse, and short-and long-term relationship) for each combination as well as facial attractiveness. In line with the sexual selection theory of music, an increase in musical performance quality was associated with higher ratings on the respective scales (with a few exceptions) for both sexes. However, the effects of facial attractiveness on the set of ratings were found to be much larger than the effect of musical performance quality. Moreover, music performance quality affected females’ ratings more than males’ ratings, whereas the latter were more influenced by facial attractiveness than the former. Taken together, the studies by Watkins (2017) and Madison et al. (2018) involving language and music skills indicate that for both sexes, cognitive and biological factors play a role in romantic attraction, and that females are more influenced by factors such as cognitive intelligence and creativity than males, for whom facial attractiveness appears to be the more relevant cue in mate choice.

Marin et al. (2017) studied the psychological mechanisms underlying the contingent effect of music on facial attractiveness and dating desirability ratings in males and females. In a crossmodal priming paradigm, excerpts of piano solo music were used as primes and other-sex faces as targets. Musical arousal and pleasantness were manipulated to test whether misattribution of arousal may underlie priming effects. Indeed, compared to a silent control condition, females reported higher facial attractiveness and dating desirability ratings after musical priming, with the highest ratings associated with high-arousing music, supporting the idea of misattributed arousal (White et al., 1981). These effects were not present among males, and the study could not reveal any significant effects of females’ cycle phase. Since high-arousing music is also more complex than low-arousing music (Marin and Leder, 2013), these results can also be interpreted within the context of Darwin’s sexual selection theory of music (see also Charlton, 2014), suggesting that cognitive and affective music-induced effects on face perception cannot be easily disentangled. Furthermore, participants were not told that the presented music had a direct relation with the target face to be rated.

### The present study

As in Marin et al. (2017), we examined in the present study the role of musicality in sexual attraction, but this time presenting the person depicted (target) as the performer of the music (prime), thus establishing a direct link. We used the same stimulus sets as in Marin et al. (2017) (i.e., piano solo music and faces of average attractiveness) and also collected facial attractiveness and dating desirability ratings as two common measures of sexual attraction. Moreover, we decided to invite only participants who reported being single to enhance ecological validity and to be more stringent than in our previous study. Since the role of the fertility cycle is difficult to evaluate in a laboratory study because of limited resources to test large samples to demonstrate small effects, we decided not to address this question in the present study. We thus considered females who were not taking hormonal contraception, were not breast-feeding and who were not having their period on the day of the experiment. Due to the fact that males and females rated other-sex faces (and thus different target faces) we decided to conduct the statistical analyses separately for each group (see Madison et al., 2018, for a similar approach). Moreover, to enable a valid comparison of results, male and female participants were carefully matched on a range of background variables, such as musical training, mood prior to the experiment and liking of the music heard in the experiment. We consider this as a strength of our study, which will allow for a clearer interpretation of our results and comparison with Marin et al. (2017).

Based on Darwin’s theory and on the results of Marin et al. (2017), we predicted that musical priming should lead to higher ratings of facial attractiveness (H1) and dating desirability (H2) in comparison to a silent control condition in females (but see Madison et al., 2018). We further predicted, for the group of females, that high-arousing (i.e., more complex music) will lead to the largest effects for both ratings (H3–H4), and that pleasantness induced by music should not play a role (H5 (main effect) and H6 (interaction between arousal and pleasantness)), based on the results of Charlton (2014), Marin et al. (2017), and White et al. (1981). For males, assuming that music is not a sexually dimorphic trait (Darwin, 1871, but see Marin et al., 2017), we generally predicted similar, but weaker, effects of musical priming on sexual attraction (H7–H12). However, in comparison to ratings given by females, female facial attractiveness should not be strongly influenced by musical priming in males (H13) (Marin et al., 2017; Madison et al., 2018).

## Materials and methods

### Participants

Based on the results reported in Marin et al. (2017), an *a priori* statistical power analysis using G*Power 3.1.9.4 was performed. The main analysis was conducted separately for males and females because the target faces differed between the two groups (see also Madison et al., 2018). Therefore, the power analysis was also conducted for each group separately. To make the present results comparable to Marin et al. (2017), our hypotheses were also tested within the framework of repeated-measures ANOVA (analysis of variance). Thus, a set of four orthogonal contrasts following our hypotheses were computed, and the power analyses were conducted for each contrast. In Marin et al.’s (2017) group of females, the average within-subject correlation across all possible correlations between the five conditions (i.e., control and four musical priming) was *r*(62) = 0.930 for attractiveness ratings and *r*(62) = 0.944 for dating desirability ratings. In the group of males, the average within-subject correlation across the five conditions was *r*(30) = 0.955 for attractiveness ratings and *r*(30) = 0.964 for dating desirability ratings.

For the current group of females, a medium effect size of 0.274 (Cohen’s *f*, directly computed from partial eta-squared reported in Marin et al., 2017), with an alpha of 0.05, a desired power of 0.80 and a correlation among repeated measure of *r* = 0.93 were used to obtain a minimum sample size for attractiveness ratings of contrast 1. The results yielded a sample size of six with an actual power of 0.82. If the within-subject correlation was lowered to *r* = 0.80, to be more conservative, the sample size increased to 13 with an actual power of 0.82. For dating desirability ratings and contrast 1, we predicted a large effect (Cohen’s *f* = 0.593), and a within-subject correlation of *r* = 0.944, which yielded a sample size of three and an actual power of 0.85. With a lower correlation of *r* = 0.80, the sample size increased to five with an actual power of 0.87. Sample sizes for contrast 2 were similarly determined by basing the computation on the exact results reported in Marin et al. (2017). For attractiveness ratings, a minimum sample size of four participants was required to reach an actual power of 0.84. For dating desirability ratings, the determined sample size was six, with an actual power of 0.89. For attractiveness ratings of contrasts 3 and 4, no significant effects (η_p_^2^ < 0.001) were expected. For dating desirability ratings and contrasts 3 and 4, the effect sizes were also negligible. For the group of males, we did not predict any significant effects on attractiveness ratings. For dating desirability ratings a marginally significant effect was observed in Marin et al. (2017) for contrast 1, and a power analysis for contrast 1 yielded a sample size of four with an actual power of 0.921.

It was decided to test a similar number of participants as in Marin et al. (2017), although the power analysis revealed that such a large sample was not necessary for testing our hypotheses. Another reason for testing larger samples was that we did not control for whether a woman was in the fertile or infertile phase of the reproductive cycle, thus to obtain an unbiased sample a larger number of participants was considered as more appropriate. The same holds true for the background variables of musical training and music preference, which are unlikely to be similar across small samples. Last, although the stimuli of the current study were the same as those used in Marin et al. (2017), this study is not a direct replication, and the criterion regarding the relationship status of the participants and the instructions were different, which further justifies testing two larger samples of participants.

Fifty-seven female heterosexual participants (mostly German and Austrian psychology students) were tested, out of which 22 were excluded prior to the exploratory data analysis. Eleven students were excluded due to technical problems (during the experiment it was discovered that one version of the computer program used to conduct the experiment had the wrong rating order in one of the two blocks). Two participants were excluded because they were using hormonal contraception, and seven were excluded because they had their menstruation on the day of the experiment. Two female participants reported more than 3 years of musical training and were thus also excluded.

Thirty heterosexual male participants were tested, and one person had to be excluded because he previously participated in a similar experiment. Three male participants reported more than 3 years of musical training, and one participant was not wearing the headphones in an appropriate way during the experiment. One male participant reported mild hearing loss in one ear, but was not excluded because the experiment was not measuring fine-grained music perception skills.

After an exploratory data analysis of the background variables, two other male participants were excluded: in order to balance the number of participants of the experimental conditions, one male participant was excluded because he reported extreme tiredness. Another male participant reported very low liking ratings of Romantic piano music (not at all comparable with ratings by female participants), and was thus excluded. It is important to note that all exclusions were made prior to the main analysis of the experimental data.

Our final two groups of 35 females and 23 males were matched across several background variables (Tables 1, 2). All participants reported being single and not in a relationship. Females were not taking hormonal contraception, not pregnant and not breast-feeding. The two groups of male and female participants did not statistically differ with respect to age, the three subscales of the multidimensional mood questionnaire, years of musical training, role of music in their life, and liking for the piano music played in the experiment (Table 1). Moreover, participant groups were similar regarding their music listening behavior and preference for classical music (Table 2). Additionally, the two groups also reported on their wish to have children in the future (5 missing values), which was similar in females (21 yes, 14 no or not sure) and males (13 yes, 5 no or not sure), χ^2^(1) = 0.77, *p* = 0.38.

**Table 1:** Participant characteristics I.

Group	Age	Mood pos.-neg.	Alertness/fatigue	Quietude/disquietude	Yrs. musical training	Role of music in life	Liking of solo piano music
Females							
*M*	22.2	17.66	14.51	16.17	0.88	5.20	4.77
*SD*	2.8	1.94	3.50	2.43	1.07	1.68	1.66
Males							
*M*	23.4	17.35	15.48	15.70	1.13	5.96	4.96
*SD*	2.9	2.27	2.91	2.67	1.20	1.22	1.15
Mann–Whitney-U							
*U*	304.50	380.00	341.50	357.00	339.50	299.50	399.00
*p*	0.117	0.717	0.329	0.465	0.361	0.091	0.955

Summary in terms of groups’ mean age, the three MDBF scores, years of musical training, role of music in life, and liking of 19th-century solo piano music. *M*, mean; *SD*, standard deviation; *U*, test statistic of the Mann–Whitney-U test; *p*, calculated probability; Yrs., years; pos., positive; neg., negative; MDBF, Mehrdimensionale Befindlichkeitsfragebogen – multidimensional mood state questionnaire. *N*_women_ = 35, *N*_men_ = 23. All degrees of freedom are 2. Role of music in life and liking of piano music were assessed on 7-point scales. All variables were used to match groups.

**Table 2:** Participant characteristics II.

Group	Listening to classical music	Active music listening	Passive music listening	Going to concerts^*^	One-night stand	Long-term relationship
Females						
*M*	2.63	4.14	5.89	3.41	3.97	3.49
*SD*	1.42	1.94	1.53	1.78	2.12	1.76
Males						
*M*	2.74	4.57	6.09	3.68	2.96	3.39
*SD*	1.32	1.59	0.90	1.49	1.92	1.64
Mann–Whitney-U						
*U*	375.50	349.00	392.00	332.00	288.00	396.50
*p*	0.657	0.388	0.859	0.474	0.065	0.922

Summary of groups’ musical listening behavior and the reported interest in a one-night stand and long-term relationship. *M*, mean; *SD*, standard deviation; *U*, test statistic of the Mann–Whitney-U test; *p*, calculated probability. *N*_females_ = 35, *N*_males_ = 23. All degrees of freedom are 2. All ratings were given on 7-point scales with low numbers indicating a lower frequency. For the variables “one-night stand” and “long-term relationship” lower numbers indicate high willingness. All variables, except for “one-night stand” and “long-term relationship”, were used to match groups.

* *N*_females_ = 34, *N*_males_ = 22.

### Materials and measures

The same materials as reported in Marin et al. (2017) were employed. For the two versions of the experiment (i.e., one for males and one for females), we used 20 male and 20 female faces of average attractiveness (Europeans with light complexion) as targets, respectively. Targets were always other-sex faces, and 17 same-sex faces were used in distractor trials. All faces were presented in frontal view, with direct gaze and neutral facial expression on a gray background (Schacht et al., 2008). Musical primes were 80 excerpts of 19th-century Romantic piano solo music (available on OSF). Since these piano excerpts were played by a solo performer, the choice of this musical style increased the credibility of the task. These excerpts had a length of 25 s and varied in their emotional contents (low vs. high arousal, unpleasant vs. pleasant), and were selected from stimuli rated in Marin and Leder (2013). Seventeen additional excerpts were used in distractor trials. Compressed file sizes, a measure of musical complexity, varied significantly between low-and high-arousing excerpts (see Marin et al., 2017, for further details on the stimuli). Facial attractiveness was assessed on a 7-point scale, ranging from (1) *very unattractive* to (7) *very attractive*, and the instructions were: *“Please report the perceived sexual attractiveness of the face.”* Dating desirability was also assessed on a 7-point scale, ranging from (1) *no, by no means* to (7) *yes, by all means*, with the following instruction: “*Please report whether you would like to date this person.”*

We used the short form A of the three-dimensional mood questionnaire [MDBF A] by Steyer et al. (1994) to assess mood, alertness and quietude prior to the experiment. In a short self-developed questionnaire after the experiment, participants reported on their socio-demographic and musical background (musical preference, listening behavior, and musical training), and their liking of the music played in the experiment (i.e., 19^th^-century piano solo music). Furthermore, participants were asked to report on their willingness to have a one-night stand or to enter a long-term relationship with the most attractive people shown in the experiment. Females were asked to provide information regarding their menstrual cycle and hormonal contraceptive use. To be specific, we asked the following questions: *Did you like the music heard in the experiment?* (1) not at all … (7) very much; *What role does music play in your life?* (1) no role … (7) a very large role; *How often do you listen to classical music?* (1) never … (7) very often; *Have you ever received musical training (university, music school, private lessons)?* Report of years of musical training and when it was finished; *How often and how do you listen to music? Passive, while doing other things:* (1) never … (7) very often; *Active, while not doing other things:* (1) never … (7) very often; *Active in a concert:* (1) never … (7) very often; *Please think of the faces that you regarded as particularly attractive. Report on your wish to have a one-night stand or a long-term relationship with these persons*. For each part: (1) very much … (7) not at all. They also reported on how difficult it was for them to judge facial attractiveness on a scale ranging from (1) very easy to (7) very difficult. Using open questions, participants were asked to report on their thoughts about the research question/hypothesis underlying the experiment. In addition, three standardized questionnaires on emotional intelligence, empathy and personality were administered but not evaluated for this study.

### Procedure

The study was approved by the local ethics committee of the University of Innsbruck, Austria (approval number 16/2019). Participants were recruited from a pool of (mostly undergraduate) psychology students. The study was advertised to investigate the perception of facial attractiveness. There was no reference to music to avoid selection bias. Students either received course credits or a monetary compensation of 12 Euro.

Participants were tested in a quiet room with constant lighting conditions and no window. The experiment was run in Matlab R2017a (The MathWorks Inc., Natick, United States) and the music was played through Sennheiser HD 380 Pro headphones at a fixed loudness level (70 dB as measured with a dB meter). Instructions and faces were presented on a 24 inch screen (Acer 24 inch, TFT display monitor) placed approximately 70 cm away from the sitting participant. After having signed the informed consent form, participants were asked to report on their mood prior to the experiment, then the actual experiment started.

The experiment consisted of two blocks (priming and control), which were counterbalanced across participants. Moreover, there were two rating scales (facial attractiveness and dating desirability), whose order was also counterbalanced across participants of the two block orders. For each participant the rating order was the same in both blocks. After a practice trial, participants began the experiment. To avoid fatigue, participants were encouraged to take a self-paced break after half of the trials were completed in the priming block.

In the (silent) control condition, participants were asked to rate 37 faces (20 targets and 17 same-sex distractor faces to prevent demand characteristics) of potential partners on facial attractiveness and dating desirability. Each trial was announced with a statement shown on the screen for 5 s (*“The next trial will follow soon”*), then the photograph followed for 2 s on a black background. After the photograph had disappeared, one of the two rating scales was shown on the screen and participants answered by mouse click. Then the other scale was shown. All faces were randomly presented.

In the priming block, the same types of ratings were obtained for each target. Each of the 20 target faces was shown for four times and randomly combined with one of the 20 musical excerpts of each emotion quadrant (spanned by arousal and pleasantness). These 80 trials were intermingled with 17 distractor trials. All trials were randomly presented and announced for 5 s with a statement saying *“The next trial will follow soon.”* While participants were listening to the music, a small white cross was shown in the middle of the screen. Participants were asked to look at it in order not to miss the onset of the visual target. Then both ratings were given before the next trial began.

The only difference between the procedure described in Marin et al. (2017) and the one used in the current experiment is a statement in the general instructions, namely that the musical excerpts were presented as played by the people shown on the photographs which followed the musical excerpts: “Dear Participant, you will now be asked to provide ratings on a series of participants who could be potential partners. In each trial you will first listen to music, which was played on the piano by the respective person. Then you will see their face. You will be asked to rate the sexual attractiveness and your willingness to have a date with this person. While listening to the music, please look at the fixation cross. Please provide spontaneous ratings.”

When the experiment was completed, participants were asked to fill in a short self-developed questionnaire. In total, the experimental session lasted around 90 min. Participants were either paid or assigned course credits, thanked and debriefed.

### Statistical analysis

All analyses were conducted using IBM Statistics SPSS 26. In the case of violations of sphericity (i.e., a significant Mauchly Test), Greenhouse–Geisser corrections were performed. Effect sizes for eta or partial eta squared were interpreted by following Cohen’s (1988) suggestions (small = 0.01, medium = 0.06, and large = 0.14, but see Lakens (2013) for limitations of this interpretation for within-subject comparisons). Within-subject error bars were computed using the *superb* package in R.^1^ When interpreting the results of orthogonal, planned contrasts, it is not necessary to observe significant main effects or interactions in the ANOVA (Gonzales, 2009).

## Results

Distractor trials (i.e., trials with same-sex faces) were not analyzed and removed from the data set. Males and females were analyzed separately because the target faces differed between groups (Marin et al., 2017). A set of four orthogonal contrasts within the framework of repeated-measures analysis of variance (ANOVA) were computed to test our hypotheses regarding attractiveness and dating desirability ratings, respectively. Contrast 1 compared the silent control condition with the average across the four musical priming conditions. Contrast 2 tested the effect of music-induced arousal and compared differences between musical primes with low-and high-arousing music. Contrast 3 compared the effect of primes with unpleasant music to those with pleasant music. Contrast 4 tested the interaction between music-induced arousal and pleasantness.

**Figure 1:**
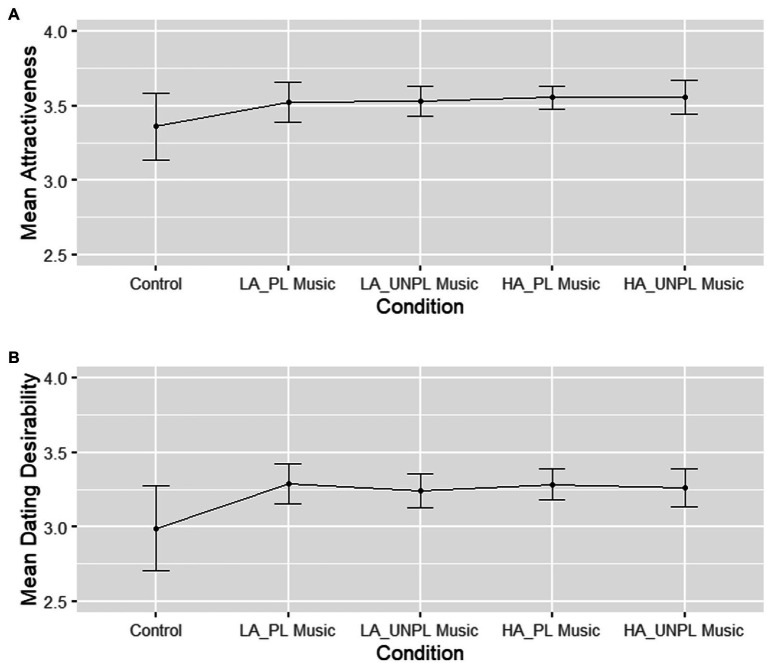
Females’ average facial attractiveness ratings of males given for each experimental condition. **(A)** Average facial attractiveness ratings. **(B)** Average dating desirability ratings. Error bars represent correlation-and difference-adjusted 95% confidence intervals. LA, low-arousing; HA, high-arousing; PL, pleasant; UNPL, unpleasant. Scales range from 1 to 7, with one indicating very low attractiveness/dating desirability.

We first tested whether attractiveness ratings were affected by musical priming in females (Figure 1A). A repeated-measures ANOVA with condition as within-subject factor (control and 4 music conditions) revealed a marginal effect of condition, *F* (2.15, 72.96) = 2.90, *p* = 0.058, η_p_^2^ = 0.08 (medium effect). The planned contrast analyses revealed that attractiveness ratings were significantly higher in the music conditions (*M* = 3.54, 95% CI [3.23, 3.85]) than in the control condition (*M* = 3.36, 95% CI [3.05, 3.67]), *F*(1, 34) = 4.44, *p* = 0.043, η_p_^2^ = 0.12 (medium effect). Ratings did not significantly differ between low-arousing (*M* = 3.53, 95% CI [3.21, 3.84]) and high-arousing music (*M* = 3.56, 95% CI [3.24, 3.87]), *F*(1, 34) = 0.53, *p* = 0.470, η_p_^2^ = 0.02 (small effect). Music-induced pleasantness did not affect attractiveness ratings, *F*(1, 34) = 0.01, *p* = 0.916, η_p_^2^ < 0.001 (no effect). There was no significant interaction between music-induced arousal and pleasantness on attractiveness ratings, *F*(1, 34) = 0.001, *p* = 0.981, η_p_^2^ < 0.001 (no effect).

Next, we analyzed dating desirability ratings and how they were affected by musical priming in females (Figure 1A). A repeated-measures ANOVA with condition as within-subject factor (control and 4 music conditions) revealed a significant effect of condition, *F*(1.78, 60.38) = 4.81, *p* = 0.014, η_p_^2^ = 0.12 (medium effect). Contrast 1 comparing the control condition to the four music conditions was significant, *F*(1, 34) = 6.45, *p* = 0.016, η_p_^2^ = 0.16 (large effect), with lower dating desirability ratings for the control condition (*M* = 2.99, 95% CI [2.63, 3.35]) than for the music conditions (*M* = 3.27, 95% CI [2.89, 3.64]). There was no significant difference between the conditions of low-and high-arousing music, *F*(1, 34) = 0.04, *p* = 0.848, η_p_^2^ = 0.001 (no effect). Similarly, there was no significant effect of musical pleasantness, *F*(1, 34) = 1.73, *p* = 0.196, η_p_^2^ = 0.05 (small effect). The interaction between music-induced arousal and pleasantness was not significant, *F*(1, 34) = 0.12, *p* = 0.733, η_p_^2^ = 0.003 (no effect).

**Figure 2:**
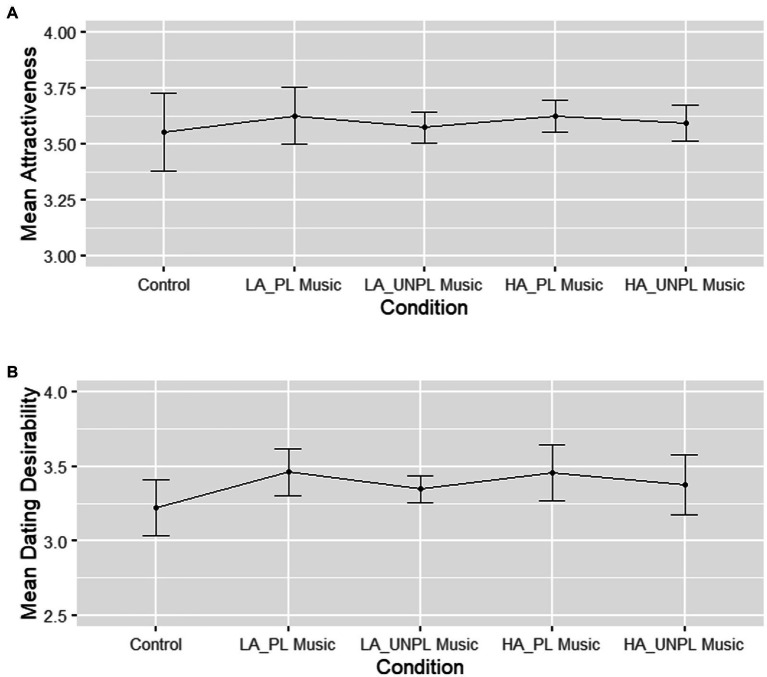
Males’ average facial attractiveness and dating desirability ratings of females given for each experimental condition. **(A)** Average facial attractiveness ratings. **(B)** Average dating desirability ratings. Error bars represent correlation- and difference-adjusted 95% confidence intervals. LA, low-arousing; HA, high-arousing; PL, pleasant; UNPL, unpleasant. Scales range from 1 to 7, with one indicating very low attractiveness/dating desirability.

In males, facial attractiveness ratings were not affected by musical priming (Figure 2A). A repeated-measures ANOVA with condition as within-subject factor (control and four music conditions) revealed no significant effect of condition, *F*(2.07, 45.62) = 0.67, *p* = 0.521, η_p_^2^ = 0.03 (small effect). Contrast 1 showed no significant difference between the silent control and the four music conditions, *F*(1, 22) = 0.57, *p* = 0.458, η_p_^2^ = 0.03 (small effect). Contrast 2 revealed that there was no difference between the low-and high-arousing conditions, *F*(1, 22) = 0.09, *p* = 0.771, η_p_^2^ = 0.004 (no effect). Contrast 3 showed no significant difference between the unpleasant and pleasant primes, *F*(1, 22) = 2.52, *p* = 0.127, η_p_^2^ = 0.10 (medium effect). Contrast 4 tested the interaction between music-induced arousal and pleasantness, which was not significant either, *F*(1, 22) = 0.16, *p* = 0.695, η_p_^2^ = 0.007 (no effect).

Finally, we examined the effects of musical priming on dating desirability in males (Figure 2B). A repeated-measures ANOVA with condition as within-subject factor revealed a marginal effect of condition, *F*(2.07, 45.45) = 2.84, *p* = 0.067, η_p_^2^ = 0.11 (medium effect). Contrast 1 was significant, *F*(1, 22) = 6.88, *p* = 0.016, η_p_^2^ = 0.24 (large effect), with higher ratings of dating desirability for the music conditions (*M* = 3.41, 95% CI [2.94, 3.88]) than for the control condition (*M* = 3.22, 95% CI [2.74, 3.71]). Contrast 2 testing for the effects of music-induced arousal was not significant, *F*(1, 22) = 0.08, *p* = 0.784, η_p_^2^ = 0.003 (no effect). Contrast 3 testing for the effects of pleasantness was not significant, *F*(1, 22) = 1.49, *p* = 0.235, η_p_^2^ = 0.06 (medium effect). Last, the interaction between music-induced arousal and pleasantness was not significant, *F*(1, 22) = 0.16, *p* = 0.697, η_p_^2^ = 0.007 (no effect).

Since the group of male participants was smaller than the group of female participants, we repeated the analysis with 25 participants including the two outliers that were removed after the exploratory data analysis. The pattern of results was very similar for both attractiveness and dating desirability ratings (see Supplementary material).

Table 2 shows that there was a marginal effect indicating that females (*M* = 3.97, *SD* = 2.12) reported to a larger degree than males (*M* = 2.96, *SD* = 1.92) that they would be willing to have a one-night stand with the most attractive persons shown in the experiment, *U* = 288.00, *p* = 0.065, *η*^2^ = 0.06 (medium effect). There was no significant difference between females (*M* = 3.49, *SD* = 1.76) and males (*M* = 3.39, *SD* = 1.64) reporting on their desire to have a long-term relationship with the most attractive persons shown in the experiment, *U* = 396.50, *p* = 0.922, *η*^2^ < 0.001 (no effect).

Participants were invited to report on their thoughts about the purpose of the experiment. All except two participants reported that the study is about the effect of music (i.e., not musicality but music experienced as sound) on attractiveness. One male and one female participant referred to the effect of musical talent (musicality) on attractiveness. Speculations about the role of music in the experiment mostly comprised mood induction (positive vs. negative) and references to effects of musical parameters such as tempo and loudness. Males and females did not differ regarding their reported difficulty in judging the facial attractiveness of faces, *U* = 398.00, *p* = 0.921, *η^2^* < 0.001 (no effect), which was generally rated as of intermediate difficulty, *M* = 4.78, 95% CI [4.34, 5.17] on a 7-point scale.

## Discussion

Darwin’s (1871) sexual selection hypothesis for the evolution of musicality has gained recent attention in the field, partly due to its clear predictions and slowly growing empirical support (e.g., Charlton, 2014; Marin et al., 2017; Madison et al., 2018; Chang et al., 2021; for a review see Ravignani, 2018; Fink et al., 2021). Musicality can be embedded into the wider context of human artisticality and creativity, for which empirical evidence for sexual selection has also accumulated (e.g., Karamihalev, 2013; Galasinska and Szymkow, 2022; Varella et al., 2022). Here, we tested Darwin’s assertion in relation to one aspect of musicality, namely instrumental music.

People use multiple cues in partner choice (Regan et al., 2000), and thus combining cues from several modalities may enable researchers to assess the effect of musicality on partner choice in a more ecologically valid setting. Here, we followed our previous approach by combining musical primes with facial targets (Marin et al., 2017). In the current study, the people shown on the photographs were presented as performers of the musical excerpts. Two groups of male and female participants, matched on several background variables, rated other-sex faces on facial attractiveness and dating desirability (two common measures of sexual attraction) after having listened to musical excerpts varying in emotional contents. The general picture emerging from our analysis is that musicality related to instrumental music may be a potential cue in mate choice in both sexes, with stronger effects in females than in males. In the group of females, H1 and H2 were supported by the data, indicating that both attractiveness and dating desirability ratings increased after music exposure compared to a silent control condition. However, there was no supporting evidence for H3 and H4 because both ratings were similar after low-and high-arousing music. H5 and H6 were supported by the data, suggesting that there was no significant main effect of music-induced pleasantness and no interaction between pleasantness and arousal. In the group of males, the results for attractiveness and dating desirability ratings differed. There were no significant effects whatsoever for attractiveness ratings (refuting H7 and H9, supporting H11), but musical priming led to higher dating desirability ratings in comparison to the silent control condition (supporting H8 and H12, refuting H10). In line with our prediction (H13), the contrasts on attractiveness ratings revealed that males’ ratings of facial attractiveness were not as strongly influenced (non-significant small effect) as those of females (significant medium effect). Females also reported to a larger degree than males to be willing to have a one-night stand with the most attractive persons seen in the experiment (marginal effect), and the sexes did not differ in their desire to have a long-term relationship with these persons. In agreement with Darwin’s theory (1871), our data suggests that having listened to short, high-quality excerpts of solo piano music increases male facial attractiveness and dating desirability in females, and dating desirability in males when rating female faces. Therefore, we found further evidence that the experience of music can alter the perception of the human face in a romantic scenario, and moreover, that music has a positive effect on a behavioral measure of sexual attraction, namely dating desirability (Marin et al., 2017; Chang et al., 2021).

Our findings are consistent with Darwin’s (1871) view that music plays a role in romantic attraction in both sexes (Madison et al., 2018), not just among females as previously reported (May and Hamilton, 1980; Charlton, 2014; Marin et al., 2017). However, our findings indicate that female facial attractiveness is impervious to musical priming in males (Watkins, 2017; Madison et al., 2018), which is probably due to its biological significance in being a marker of fecundity (Johnston, 2006). At the same time, males’ behavior may still be affected by indicators of musicality (see also Madison et al., 2018), which clearly warrants further experiments on sex differences regarding how different cues affect various measures of partner choice (Jonason et al., 2012; Walter et al., 2020). More generally, our results can be interpreted within the context of the mutual mate choice (MMC) model, which accommodates both the fact that sexual dimorphism is relatively low among humans and that there are still some evolved sex differences (Miller, 2013; Stewart-Williams and Thomas, 2013). Both sexes have artistic motivations, capacities for appreciation and production of music, but there are still some differences in the degree and circumstances under which these tendencies are expressed (Varella et al., 2017). Mutual mate choice is necessary because in humans there is high paternal investment and alloparental care (e.g., grandmother effect), so both sexes compete for partners and both sexes select mates.

Contrary to our expectations, and not in line with Darwin’s theory, high-arousing, more complex music did not yield the largest effects on sexual attraction (but see Charlton, 2014; Marin et al., 2017; Madison et al., 2018). This discrepancy between research findings may be attributed to differences in experimental designs and research questions. First, the within-subject design of the present study clearly differs from a design in which a face is only paired once with a musical excerpt varying in affect and complexity (Madison et al., 2018) or from a two-alternative forced-choice task in which two musical excerpts varying in complexity are presented without any visual stimuli (Charlton, 2014). There are advantages and disadvantages to these designs, but ours enabled us to show that music has an effect on face perception and dating desirability in the first place because we employed a silent control condition (which was not present in the designs of Charlton, 2014, and Madison et al., 2018). Future studies may also add other types of control conditions besides silence. Second, in Marin et al. (2017), misattribution of arousal, as an unconscious mechanism explaining the effect of music-induced affect on face perception, was the focus of interest. Considering that only the instructions differed between Marin et al.’s (2017) and the present study, it is likely that by establishing a cognitive, conscious link between musical primes and faces, the participants’ attention was guided to different aspects of the musical excerpts than in Marin et al. (2017). In the current within-subject design, all musical excerpts were generally of a high standard (CD recordings of world-class performers) and allegedly stemmed from the same performer in the four music conditions. Thus, the general positive impression associated with the performer may have overruled the likely, more subtle, role of the varying affective and compositional contents across these excerpts.

Interestingly, Figures 1B, 2 show that music-induced pleasantness, and not arousal, plays a non-significant role in determining sexual attraction, especially in males and for ratings of dating desirability. We interpret this observation by suggesting that induced pleasantness in a mating context may be perceived as rewarding and thus elicit approach behavior, which would be in line with Darwin’s argument that “the progenitors of man, either the males or females or both sexes, before acquiring the power of expressing their mutual love inarticulate language, endeavored to charm each other with musical notes and rhythm” (1871, p. 880). Throughout his book *The descent of man* Darwin stresses the charming character of sounds during mate choice in animals and humans, which spurs the discussion about the mutual role of affective and cognitive cues in musical signals. Musical complexity as a signal of advanced motor skills, intelligence, and creativity may not be the only way through which music affects romantic attraction (Marin et al., 2017). There may also be an additional (or alternative) route, possibly including both affective and aesthetic responses, as suggested by Darwin. To be specific, Darwin explained the evolution of ornaments by their inherent aesthetic quality and not necessarily by which fitness quality they could indicate. There has been an ongoing theoretical debate about the exact mechanisms underlying sexual selection (Miller, 1998; Davis and Arnocky, 2022). Thus, future behavioral studies should be more carefully designed to be able to differentiate between such mechanisms.

At present the role of individual differences in musical priming effects on sexual attraction was not examined. Previous research has shown that subjective experience of music-induced complexity and arousal depends on person-related factors such as personality traits and musical background in females (Marin and Leder, 2018). Although the current sample of non-musicians and the one of Marin et al. (2017) did not differ regarding a wide range of background variables including musical training, age and mood, the sample of the current study listened less frequently to classical music and liked the piano solo music of the experiment less than the sample of Marin et al. (2017). This may explain why the effects of music-induced arousal and pleasantness were less differentiated in the current study than expected. Interpersonal attraction is partly determined by the similarity-attraction effect (Caspi and Herbener, 1990; Tidwell et al., 2013), thus future studies will have to shed light on the extent to which individual differences regarding musical sophistication and one’s self-perception of musicality may impact on the mechanisms determining the power of musicality in mate choice.

There are several limitations of the present research that need to be addressed. At the moment, our findings are not generalizable beyond the Western population studied in the experiment (Henrich et al., 2010), thus cross-cultural studies should be conducted as a next step. The current study focused on sexual attraction in the context of short-term mating. However, as Charlton (2014) found, we may expect differential effects of musicality on short- vs. long-term mating strategies in females. In this regard, it may also be worthwhile to include a wider range of person-related rating scales (see Madison et al., 2018). Another limitation concerns the present focus on motoric (performance) skills as an indicator of musicality. The role of mental fitness indicators in partner choice, such as creative intelligence as seen in musical improvisations (Madison et al., 2018), may also be studied in combination with biological cues in the future. Given that we studied music pertaining to a single musical style, our findings should not be over-interpreted but taken as a motivation to extend this research program to other musical styles and listener groups.

Our empirical findings have important implications for a better understanding of mating behavior observed in real-life settings in which music and dancing plays a role. For example, nightclubs have been identified as human mating grounds (Mannion et al., 2009) in which background music might be one factor influencing the sexual appeal of others. The positive effect of background music on dating desirability has recently been demonstrated by a speed dating paradigm (Chang et al., 2021). Moreover, the socio-cultural phenomenon of groupies searching for sexual attention of rock stars (Barres, 2005; Larsen, 2017) as well as the phenomenon of adolescent girls seeking platonic romantic passions with male musicians and other celebrities (Engle and Kasser, 2005) may be partly based on perceptual crossmodal interactions.

In conclusion, crossmodal priming paradigms may be useful to study the evolution of music from a psychological and behavioral perspective. We show that being exposed to music may increase sexual attraction in both sexes when rating faces of average attractiveness. In light of growing evidence for Darwin’s sexual selection theory, we will need to discuss whether his theory should stand on its own, or whether it should be integrated into broader, adaptationist theories which include other aspects of social bonding, such as singing to infants and social grooming (Savage et al., 2020; Leongómez et al., 2021).

## Data availability statement

The dataset and musical stimuli presented in the study can be found at: OSF: https://osf.io/dp3es/?view_only=6a3d15fad004479bae839dc8c9efcf79.

## Ethics statement

The studies involving human participants were reviewed and approved by the Board for Ethical Questions in Science of the University of Innsbruck. The patients/participants provided their written informed consent to participate in this study.

## Author contributions

MM developed the study concept and the study design. Testing and data collection were performed by IR and MM. IR performed the data analysis and interpretation under the supervision of MM, and provided critical revisions. MM drafted the manuscript. All authors contributed to the article and approved the submitted version.

## Funding

Our work was funded by the Tiroler Wissenschaftsförderung grant number UNI-0404/2418. Open access funding provided by University of Vienna.

## Conflict of interest

The authors declare that the research was conducted in the absence of any commercial or financial relationships that could be construed as a potential conflict of interest.

## Publisher’s note

All claims expressed in this article are solely those of the authors and do not necessarily represent those of their affiliated organizations, or those of the publisher, the editors and the reviewers. Any product that may be evaluated in this article, or claim that may be made by its manufacturer, is not guaranteed or endorsed by the publisher.

## Footnotes

^1^ https://CRAN.R-project.org/package=superb
