# Supplementary material for: Darwin’s sexual selection hypothesis revisited: Musicality increases sexual attraction in both sexes
Source: Front Psychol. 2022 Aug 25;13:971988. doi: 10.3389/fpsyg.2022.971988 (PMC9453251; doi:10.3389/fpsyg.2022.971988)
Supplement: Supplementary file 1 [file Table_1.DOCX]

Supplementary Material

# Supplementary Results

Since the group of male participants was smaller than the group of female participants, we repeated the analysis with 25 participants including the two outliers that were removed after the exploratory data analysis. For attractiveness ratings, a repeated-measures ANOVA with condition as within-subject factor revealed no significant effect of condition, *F*(1.94, 46.43) = .35, *p* = .842, η_p_² = .01 (small effect), and no significant contrasts, all *p*s > .133. For dating desirability ratings, a repeated-measures ANOVA with condition as within-subject factor revealed a significant effect of condition, *F*(2.06, 49.51) = 3.19, *p* = .048, η_p_² = .12 (medium effect). Contrast 1 was significant, *F*(1, 24) = 8.25, *p* = .008, η_p_² = .26 (large effect) and all other effects non-significant, all *p*s > .264.
